# Book Reviews

**Published:** 1872-01-15

**Authors:** 


### Book Reviews.

A Treatise on Human Physiology. Designed for the use of Students and Practitioners of Medicine, by John C. Dalton, M.D., Prof. of Physiology in the College of Physicians and Surgeons, New York, etc. Fifth edition, revised and enlarged. Henry C. Lea, Philadelphia, 1871. Chicago: For sale by Cobb, Andrews & Co.

We are happy to announce the issue of a new and revised edition of this valuable standard work. It still maintains its high reputation and deserved popularity, both as a text book and a work of reference. One of the first books that comes before him as a student, it is almost the last volume that, in after years in the busy rounds of practice, the physician would willingly spare from the shelves of his library, its well-worn and long familiar pages seeming to possess an ever fresh and recurring interest.

The very considerable advances and changes made in many departments of Physiology during the past few years, when the progress of physiological research and special investigation seems to have been especially active, rendered a careful revision of the work necessary. This revision has been faithfully carried out, and while the general plan and arrangement has not been materially altered, it has been modified and re-arranged in many of its parts, the aim of the author having been to make the book in its present form, a faithful exponent of the actual condition of physiological science at the present time.

Neuralgia, and the Diseases that resemble it, by Francis E. Anstie, M.D. New York: D. Appleton & Co. For sale by W. B. Keen & Cooke, Chicago.

This is a small volume of 350 pages. The first 220 pages are devoted to a very full consideration of Neuralgia proper, its Clinical History, Pathology, Diagnosis, Prognosis and Treatment, while Part II, constituting the remainder of the volume, is occupied with short sketches of the *principal diseases resembling neuralgia*. These latter the author gives as a kind of gallery of spurious neuralgias, in order, as he says in the *preface*, to set the diagnosis of true neuralgia from its counterfeits in the clearest light possible, and thus prove that it is not a mere offshoot of the gouty or rheumatic diatheses, and still less a mere chance symptom of a score of different and incongruous affections.

On the Treatment of Pulmonary Consumption by Hygiene, Climate and Medicine, in its connection with Modern Doctrines, by James Henry Bennet, M.D., etc. (second edition.) D. Appleton & Co., New York, 1872.

This is a concise, practical little treatise, the chapters devoted to the subjects of climatic influences, and the modern treatment of Phthisis and its results being especially valuable and intersting. In chapter VI a number of cases are given illustrative of the cure and arrest of Phthisis.

Pulmonary Consumption; its Nature, Varieties and Treatment, with an analysis of one thousand cases to exemplify its duration, by C. J. B. Williams, M. D., F. R. S., etc., and Charles T. Williams, M. A., M. D., etc. Philadelphia: H. C. Lea, 1872. For sale by S. C. Griggs & Co., Chicago.

This work includes a full and extended consideration of the pathology of consumption. There are also several chapters devoted to abstracts of cases illustrating the nature, varieties and treatment of pulmonary consumption, as also its duration. The last six chapters, occupied with the treatment of phthisis, contain full directions in regard to medicine, dietetic and hygienic measures, climate, etc.

Modern Medical Therapeutics, a compendium of Recent Formulæ and specific Therapeutical Directions, by Geo. H. Nepheys, A. M., M.D. Third edition—revised and improved. Philadelphia: S. W. Butler, publisher, 1871.

The American Practitioner, a monthly journal of Medicine and Surgery, edited by David W. Yandel, M.D., and Theophilus Parvin, M.D. Louisville: John P. Morton & Co., publishers.

We acknowledge the receipt of two handsomely bound volumes of this valuable journal, Vols. III and IV, comprising the monthly issues for the past year.

The Federal Government, its Officers and their Duties, by Ransom H. Gillet. Woolworth, Ainsworth & Co., New York and Chicago, 1871.

